# A case of symmetric retrograde thromboembolic cerebral infarction in an 8-year-old child due to arterial thoracic outlet syndrome

**DOI:** 10.1007/s00381-018-3911-x

**Published:** 2018-07-17

**Authors:** Jolanta Strzelecka, Tymon Skadorwa, Milena Franckiewicz, Sergiusz Jóźwiak

**Affiliations:** 1 Department of Pediatric Neurology, Medical University of Warsaw, Warsaw, Poland; 2 Department of Pediatric Neurosurgery, Bogdanowicz Memorial Hospital for Children, Nieklanska St. 4/24, 03924 Warsaw, Poland; 3 Department of Descriptive and Clinical Anatomy, Medical University of Warsaw, Warsaw, Poland

**Keywords:** Pediatric stroke, Thoracic outlet syndrome, Cervical rib, Retrograde thromboembolic phenomenon

## Abstract

Arterial type of thoracic outlet syndrome belongs to the most unusual mechanisms of stroke in children in the first decade of life. We present a case diagnosed for bilateral and symmetric changes due to retrograde thromboembolic phenomenon. Regarding the age of the patient, the appropriate diagnostics and management are still a matter of debate in pediatric and neurological literature.

## Introduction

Arterial thoracic outlet syndrome (aTOS) is an extremely rare cause of embolic stroke in children [4]. A symptomatic compression of the subclavian artery (SA) may result from the osteo-musculo-vascular conflict provoked by a supernumerary rib, arising from C7 vertebra [11]. It usually presents with symptoms secondary to SA stenosis and subsequent antegrade embolization, such as loss of peripheral pulse, paresthesias in the hand, claudication, or digits ischemia [15]. Retrograde thromboembolic phenomenon may reveal during overhead abduction of the arm, which favors the occlusion of SA and promotes the occurrence of carotid or vertebrobasilar stroke [2, 6].

Several papers have been published about the symptomatology of retrograde stroke, some of them refer to pediatric population, but none of reported cases presented with symmetric infarctions due to aTOS in the first decade of life.

## Case report

**Fig. 1:**
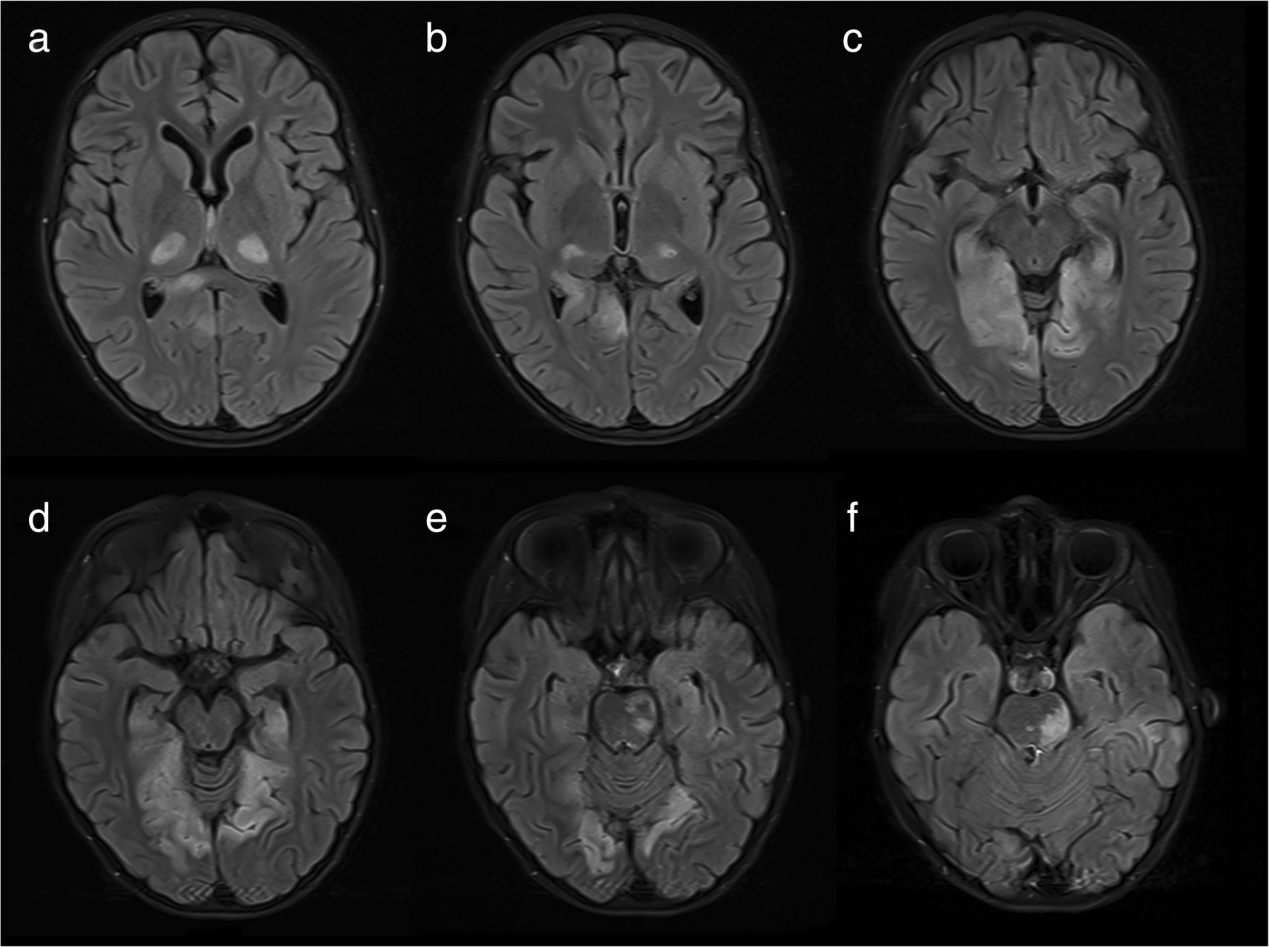
T2-weighted dark fluid MR scans show symmetrical hyperintense areas in both thalami (**a**, **b**) and medial parts of occipital lobes (**c**, **d**, **e**). Note left-sided pontine hyperintensivity (**e**, **f**) and involvement of splenium of corpus callosum (**a**)

An 8-year-old girl presented with a history of sudden morning numbness of right limbs, headache, and vomiting, followed by tonic-clonic seizures and a loss of consciousness. On admission she was somnolent, moderately dehydrated, with right hemiparesis and right hemihypoesthesia. The warmth of right limbs was decreased but the pulse on peripheral arteries was normal. Magnetic resonance (MR) imaging revealed symmetrical changes in postero-lateral thalami and medial occipital lobes. Smaller areas were noted in the region of splenium of corpus callosum and within deep structures of the left cerebral hemisphere (Fig. 1a–d).

A series of tests towards the diagnosis of metabolic, autoimmune, and rheumatoid diseases, or coagulopathies was performed. None responded positive.

**Fig. 2:**
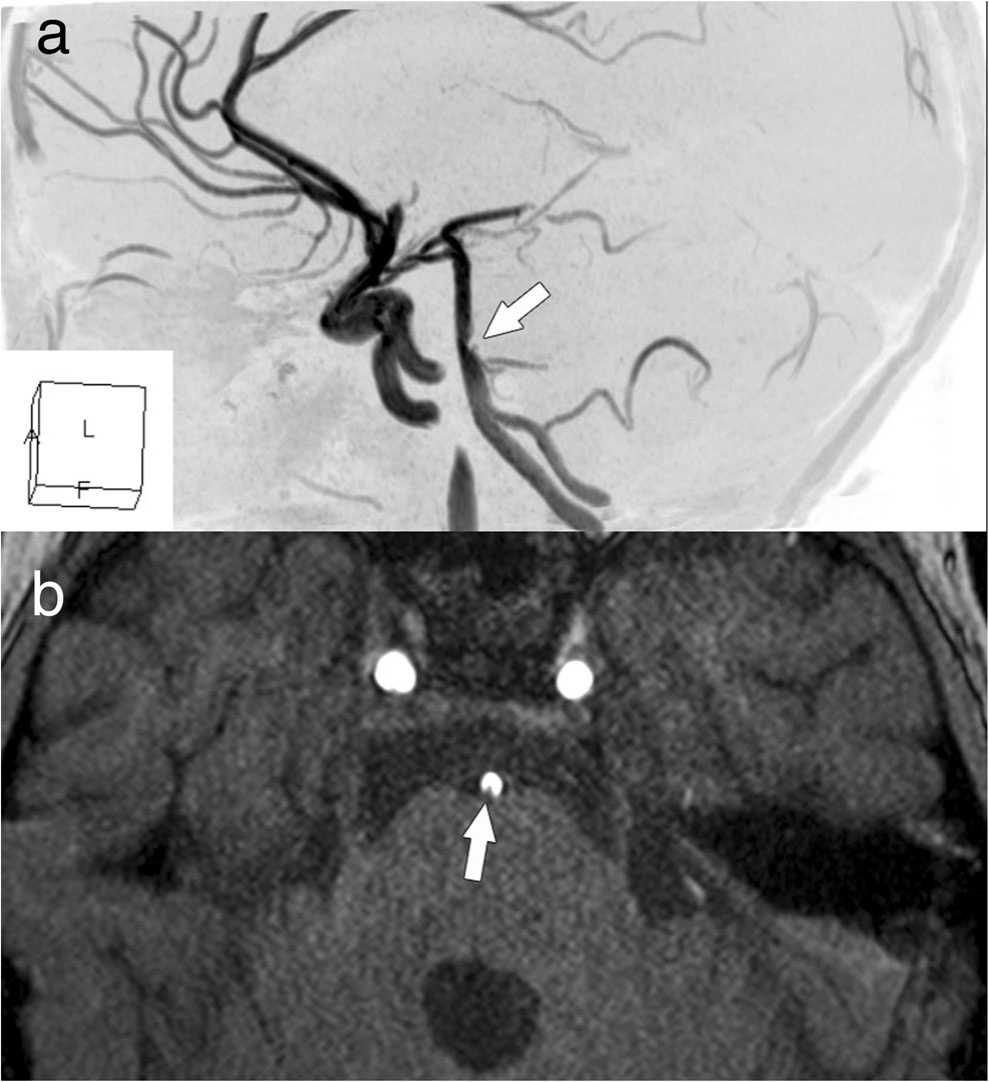
3D-TOF angiography: a lateral aspect (**a**) shows the loss of contrast medium (arrow) within the basilar artery; a horizontal aspect (**b**) presents an embolic mass within the lumen of BA (indicated by an arrow)

After 7 days, she deteriorated: became non-responsive, on neuro-exam anisocoria R > L, right-sided central facial palsy, bilateral hemiparesis R > L, and positive bilateral Babinski sign were noted. MR scan revealed a new large hyperintense area in the pons and some smaller in the cerebellum (Fig. 1e, f). 3D-TOF angiography showed an embolic mass within the basilar artery (BA) at the level of left AICA and partial occlusion of P2a segments of both posterior cerebral arteries (PCAs) (Fig. 2).

**Fig. 3:**
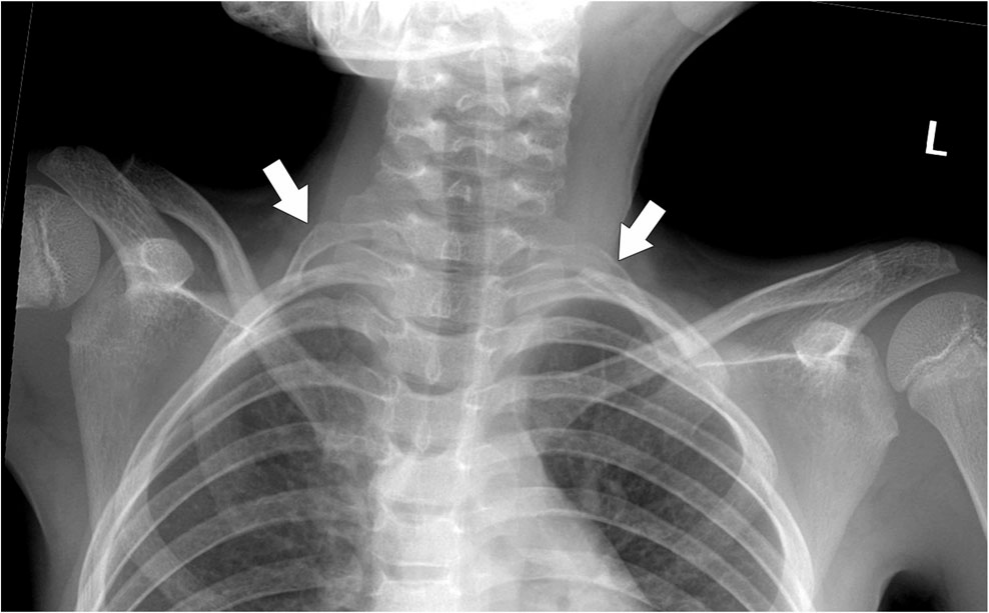
Chest X-ray demonstrating bilateral cervical ribs (indicated by the arrows)

Physical examination revealed a loss of right radial pulse. Doppler-US showed normal flow values in the arteries of right arm and forearm but the complete occlusion of right SA due to an embolic mass at the origin of right vertebral artery (VA). The VA was partially occluded but had a torticuous canal of patency in its initial segment. Chest X-Ray revealed the presence of cervical ribs bilaterally (Fig. 3).

**Fig. 4:**
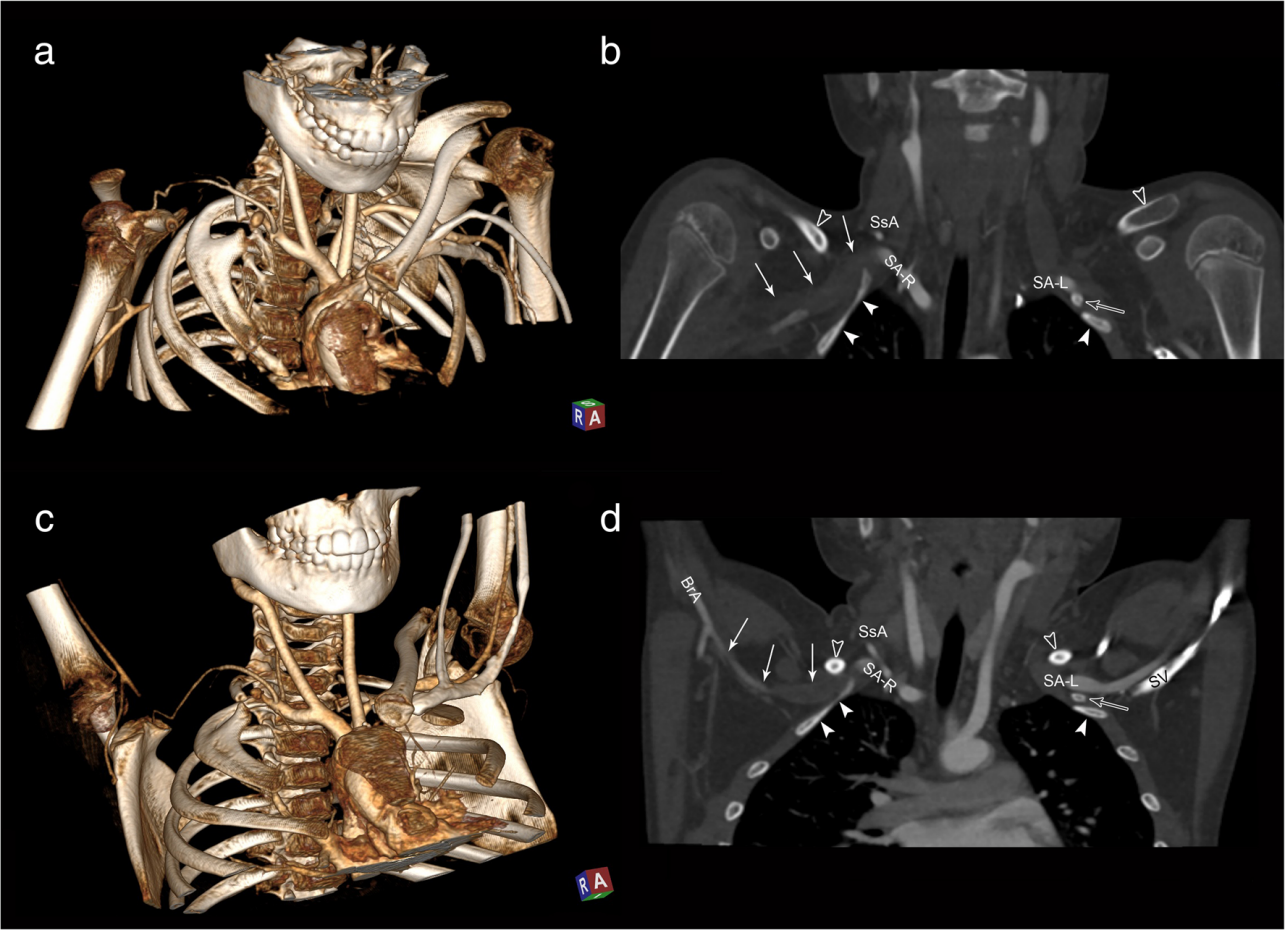
Contrast-enhanced CT scans at two arm positions (**a**, **b**—anatomical position; **c**, **d**—overhead abduction). Right clavicle removed from 3D reconstructions (**a**, **c**) for visualization of cervical-1st rib complex. Coronal cuts (**b**, **d**) show subclavian arteries (right, SA-R; left, SA-L) passing between the clavicle (empty arrowhead) and first rib (white arrowheads). Occlusion within right SA (white arrows) reaches the right brachial artery (BrA), supplied by right suprascapular artery (SsA). Contrast medium visible on the right within the proximal SA and SsA. Empty arrow shows left cervical rib. Note the distance between the clavicle and 1st rib on both sides

A CT-angiography of subclavian arteries in two typical arm positions was done (Fig. 4). It showed the occlusion of right SA with a developed suprascapular arterial anastomosis supplying the brachial artery. The scans also revealed bony anomalies in the region of superior thoracic aperture: cervical ribs and bifid 1st rib on the right, which fixed the diagnosis of right-sided aTOS, confirmed subsequently in catheter angiography a few days after. Regarding her age, surgeons decided to postpone surgical excision of cervical rib until the end of skeletal growth. She was placed on anticoagulant therapy with low molecular weight heparin combined with warfarin. After a few days, her neurological status improved—she was in good logical contact, responsive, with scattered speech. After rehabilitation sessions, she started to sit and walk without support. She was discharged with right-sided weakness and intention tremor.

## Discussion

Symmetric cerebral infarction is uncommon in pediatric population. Various inflammatory and non-inflammatory vasculopathies (moya-moya, dissecting arteriopathies, infectious arteritis) may result in stroke but they rarely cause bilateral changes [8, 9, 12, 18, 21]. The ischemic area is usually supratentorial, in a carotid distribution, especially when resulting from aTOS provoked by a right cervical rib [10, 11, 15]. Consequently, symmetric changes are considered as typical rather for metabolic or inflammatory diseases of young age, requiring a wide range of diagnostic work-up [14]. The diagnostics is primarily directed towards systemic disorders, such as coagulopathies (responsible for young age stroke in up to 50%, specifically with such risk factors as elevated lipoprotein level, homocysteinemia, factor V Leiden trombophilia, protein C/S deficiency, or antiphospholipid syndrome) or cardiac diseases which are the cause of 10–30% of pediatric stroke [7, 12, 18].

Although demonstrated by other authors, anatomical conditions are seldom considered the first-line factors promoting cerebral infarction in a young. The youngest patient with a stroke due to aTOS described so far was 14 years old and presented with right-sided hemispheric stroke combined with cerebellar infarctions [11]. Our patient also suffered from supratentorial and infratentorial infarctions but of purely vertebrobasilar distribution. This is typical for the retrograde thromboembolic phenomenon, resulting from compression of SA distal to VA origin. This mechanism explains partial occlusion of BA resulting in pons infarction and subsequent thalamic and medial occipital strokes due to obliteration of both PCAs [16].

Cervical ribs are found in less than 1% of general population (more commonly in females) and in 80–90% of cases are asymptomatic [10]. Chronic compression of SA is diagnosed in < 1% of TOS [10, 15]. When symptomatic, the treatment includes a surgical excision of a cervical rib, commonly with the first rib. This method is relatively safe and provides good functional outcome for distal complaints [1, 5, 13].

However, to date, no clear evidence on the success rate of such treatment in aTOS children with stroke has been provided. Reported pediatric series [3, 13, 17, 19, 20] contain only single aTOS cases younger than 10 years. Although surgery seems effective for distal (forearm, hand) complaints, the risks and benefits of vascular reconstruction of the potential SA aneurysm are still a matter of debate. Therefore, regarding the lack of supportive data, it seems that non-operative treatment may be considered an option for preadolescents and surgery offered until the end of skeletal growth, what favors girls after the first menstruation and boys at the age of 14–16.

## Conclusions

Symmetric cerebral infarctions are an uncommon presentation of aTOS in the first decade of life. Since retrograde embolic phenomenon may mimic inflammatory or metabolic disease, it should be of importance to exclude this rare cause of stroke. The excision of a supernumerary rib, although effective in adolescents, may be of limited benefit in preadolescent patients what favors non-operative management in case of lack of indications for urgent surgery.
